# Gut Microbiome and Myalgic Encephalomyelitis/Chronic Fatigue Syndrome (ME/CFS): Insights into Disease Mechanisms

**DOI:** 10.3390/ijms27010425

**Published:** 2025-12-31

**Authors:** Ralitsa Nikolova, Deyan Donchev, Katya Vaseva, Ivan N. Ivanov

**Affiliations:** 1 Department of Medical Microbiology and Immunology—“Prof. Dr. Elissay Yanev”, Faculty of Medicine, Medical University of Plovdiv, 4002 Plovdiv, Bulgaria; ralitsa.nikolova@mu-plovdiv.bg (R.N.); katya.vaseva@mu-plovdiv.bg (K.V.); 2 Division of Innovative Diagnostic Methods, Research Institute at Medical University of Plovdiv, 4002 Plovdiv, Bulgaria; 3 Laboratory of Microbiology, University Hospital St. George, 4002 Plovdiv, Bulgaria; 4 Strategic Research and Innovation Program for the Development of Medical University of Plovdiv (SRIPD-MUP), 4002 Plovdiv, Bulgaria; deyandonchev@ncipd.org; 5 National Reference Laboratory for Control and Monitoring of Antimicrobial Resistance, Department of Microbiology, National Center of Infectious and Parasitic Diseases, 26 Yanko Sakazov Blvd., 1504 Sofia, Bulgaria

**Keywords:** chronic fatigue syndrome (CFS), myalgic encephalomyelitis (ME), ME/CFS, gastrointestinal microbiome, gut dysbiosis, gut–brain axis, therapy

## Abstract

Myalgic Encephalomyelitis/Chronic Fatigue Syndrome (ME/CFS) is a disabling clinical condition, whose hallmark characteristic is post-exertional malaise (PEM). It can affect many organs and systems, leading to severe impairment of patients’ quality of life. Although numerous post-infectious, immunological, neurological, metabolic, and endocrine alterations have been documented, neither a definitive diagnostic marker nor approved treatments are available. The etiology and pathophysiology remain incompletely understood; however, emerging evidence suggests that the gut microbiome plays a role in immune responses and the development of ME/CFS. It is hypothesized that specific disturbances in gut microbiome composition, known as dysbiosis, may compromise the integrity of the intestinal barrier. This consequently leads to translocation of microbial components, which further triggers an immune response and systemic inflammation complicating the clinical presentation of ME/CFS. Furthermore, in terms of the so-called gut–brain axis, microbiome changes may lead to distinct neurocognitive impairments observed in ME/CFS patients. This review offers the readers a broad perspective on the topic on ME/CFS, with a particular emphasis on the interplay between the gut microbiome and disease mechanisms. Last but not least, recent data on potential treatment strategies for intestinal dysbiosis in ME/CFS patients have been included.

## 1. Introduction

Myalgic Encephalomyelitis (ME), also known as Chronic Fatigue Syndrome (CFS) or ME/CFS, is a serious and debilitating clinical condition that can last for many years [1]. It is characterized by severe, persistent, and relapsing fatigue that continues for more than six months and impairs the normal daily activities of people who are affected [2,3]. Apart from the profound exhaustion, ME/CFS patients report experiencing a variety of other nonspecific symptoms such as generalized pain, sleep disturbances, flu-like symptoms, joint and muscle pain, anxiety, problems with concentration, thinking and memory, orthostatic intolerance, gastrointestinal (GI) complaints and many others [4,5]. ME/CFS can severely influence individuals’ quality of life, leaving up to 75% of the affected people unable to work and about 25–29% home- or bedbound [6,7]. This condition is known to be more disabling than a lot of other chronic illnesses, including multiple sclerosis, stroke, lung cancer, chronic kidney disease, and type 2 diabetes [6,8]. The etiology and pathophysiology of ME/CFS remain unknown, and the lack of a specific, diagnostic test or biomarker further complicates its diagnosis [9,10]. It has been suggested that many cases may be triggered by infectious agents, with viral pathogens being the most frequent cause [11,12,13,14]. This explains why the COVID-19 pandemic has caused a significant increase in ME/CFS cases, as approximately 87% of patients who recover from acute infection with SARS-CoV-2 report symptoms similar to those of ME/CFS [15,16].

Microbiome and its role in health and disease have gained much attention in recent years. Research in that field has advanced considerably in the last two decades, with the development of new genetic techniques that enable scientists to analyze the composition and function of microbiomes in different body sites [17]. Disruptions in the gut microbiome, especially, have been linked to altered host metabolism, immune responses, and inflammation, thus offering us a new way to understand many common diseases such as cardiometabolic disorders, inflammatory bowel disease (IBD), neuropsychiatric diseases, and cancer [18,19].

The role of the gut microbiome in ME/CFS has been extensively discussed nowadays, as GI problems, particularly irritable bowel syndrome (IBS), are frequently reported by these patients, and 35% of the affected take medications to alleviate these complaints [20]. Recent data have shown the potential influence of gut health in ME/CFS pathogenesis, as gut dysbiosis is known to contribute to fatigue exacerbation and cognitive problems [21]. Shifts in the gut microbiome composition are believed to play a role in systemic inflammation, immune dysfunction, altered neurochemical signaling, and neuronal health through the dysregulation of the gut–brain axis. A better understanding of the interplay between microbiome changes and ME/CFS may shed light on the disease pathogenesis. This will further contribute to the improvement of the diagnostic process and more effective treatment strategies to enhance the quality of life of the affected individuals [22].

This review aims to synthesize the current state of knowledge from the specialized literature by providing an overview of ME/CFS case definition and diagnostic criteria, proposed etiologies, clinical manifestations, and the pathophysiological mechanisms underlying this heterogeneous condition, with particular emphasis on the interplay between the gut microbiome and ME/CFS. We aim to identify specific dysbiotic patterns that correlate with disease symptoms and severity and to highlight the possible pathways that link gut dysbiosis to ME/CFS symptoms. Finally, a brief insight into potential treatment strategies for microbiome modulation is provided.

## 2. Search Strategy

To conduct this narrative review, a comprehensive search of peer-reviewed articles written in English was performed across multiple databases including PubMed, Scopus, Web of Science and Google Scholar. A search strategy combining Medical Subject Headings (MeSH) and free-text keywords was used to ensure the inclusion of appropriate studies that explore the gut microbiome and its role in ME/CFS. The search was done with the use of Boolean operators in order to combine relevant terms such as (“gut microbiota” OR “gut bacteria” OR “gut flora” OR “intestinal bacteria” OR “intestinal flora” OR “gut–brain axis” OR “microbiota-gut–brain axis”) AND (“gut dysbiosis” OR “dysbiosis”) AND (“chronic fatigue syndrome” OR “myalgic encephalomyelitis” OR “ME/CFS”). Recent publications were preferred, but no limiting period was imposed in our screening. Studies were selected based on their title, abstract, and overall relevance. In addition, the reference lists of included studies and key review articles were manually searched to identify further relevant literature.

## 3. An Overview of ME/CFS

### 3.1. History of Case Definition

Illnesses similar to ME/CFS have been described in the literature for more than two hundred years [23]. The term that appeared first in 1959 was “benign myalgic encephalomyelitis” and was used to characterize an inflammatory disease with severe muscle pain, sensory, and cognitive symptoms [24]. CFS was introduced in 1988 by Holmes, who used it in order to describe the chronic Epstein–Barr virus syndrome, thus proposing the first case definition of that illness [25]. A few years later, Fukuda and colleagues [26] developed a revised case definition for CFS, which included the presence of chronic fatigue lasting at least 6 months as a diagnostic symptom [27]. Since the introduction of the Canadian Consensus Criteria in 2003, the combined term ME/CFS has been adopted worldwide [28]. Since then, several consensus criteria (the International Consensus Criteria [29], Institute of Medicine Criteria [24], and NICE guideline [30]) have been established to aid in the diagnostic process, as ME/CFS currently lacks a defined blood marker of diagnostic significance. This means that the diagnosis of ME/CFS relies mainly on detailed medical history, and physical and mental examination, which usually involves multiple visits to healthcare professionals. Diagnostic tests and specialist consultations are mainly done to rule out other accompanying illnesses before confirmation of ME/CFS [31].

### 3.2. Epidemiology

The global prevalence of ME/CFS differs among studies, as it depends on the case definition criteria employed for diagnosis [32]. A meta-analysis by Lim et al. reported an estimated number of nearly 0.9% of the world’s population suffering from ME/CFS when the most commonly used case definition of Centers for Disease Control and Prevention’s (CDC)-1994, known as Fukuda criteria, is applied for diagnosis [7]. ME/CFS can affect people of all ages, genders, races, and socioeconomic backgrounds, but it is more frequently described in women [24]. In most reports, illness onset is around middle ages, although there are also cases of affected children as well as older adults [33]. Community-based studies in the United States show that African American and Hispanic patients are more frequently affected by this disorder [34].

### 3.3. Clinical Manifestations

**Figure 1:**
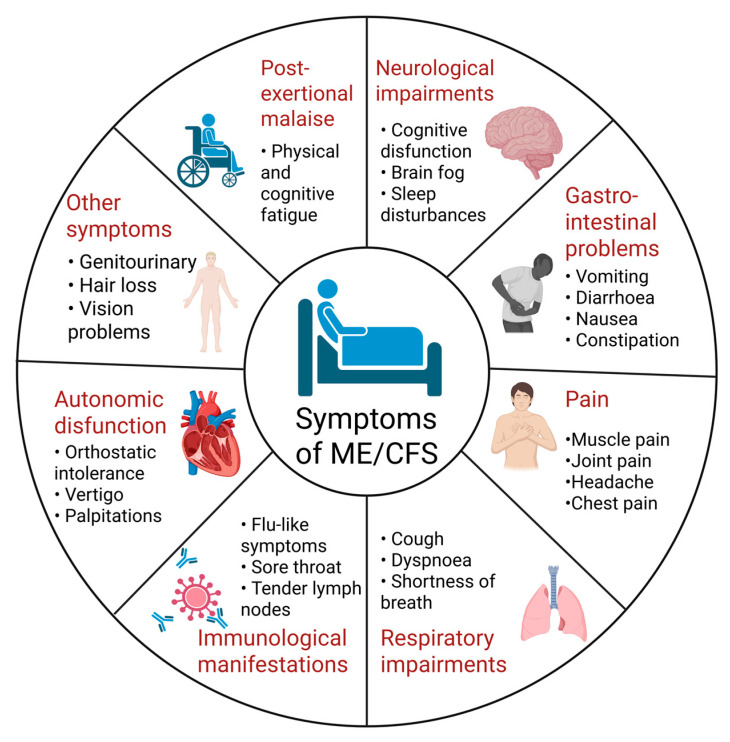
Overview of common clinical manifestations of ME/CFS [15,31,32,38]. Created in BioRender. Nikolova, R. (2025) https://BioRender.com/ob3cwtb, accessed on 1 November 2025.

ME/CFS is a condition that manifests with a variety of somatic and cognitive symptoms, which give evidence of multiorgan involvement during the disease course [30]. Although many of the clinical manifestations can overlap with those of other illnesses, there is one hallmark feature that can distinguish ME/CFS [35]. It is known as post-exertional malaise (PEM) and is defined as an exacerbation of a patient’s baseline symptoms after physical, cognitive, orthostatic, or emotional exertion [36]. One of the main characteristics of PEM is a profound exhaustion that makes patients seek complete rest in order to gain any relief. Additionally, cognitive difficulties and neuromuscular complaints, such as muscle pain and weakness, are not unusual after an event that triggers PEM [37]. This feature helps clinicians to differentiate ME/CFS from psychiatric disorders like depression, where patients typically feel better after physical activity [38]. The pattern of onset of PEM symptoms can also vary among patients, as in some of them, symptoms may appear instantly, but in others, they can be delayed by more than 24 h after the triggering event [39]. Other symptoms that are frequently reported by individuals with ME/CFS include flu-like symptoms (sore throat, enlarged lymph nodes, and periodic low-grade fevers), unrefreshing sleep, GI problems (diarrhea, constipation, and nausea), autonomic dysfunction, generalized pain, and hypersensitivity to light, sound, particular fragrances, or food [32,40,41]. The most common symptoms of ME/CFS are summarized in Figure 1. Along with the heterogeneous clinical manifestations, disease progression differs enormously between patients: while ME/CFS is usually a chronic condition, some patients may experience periods of partial recovery in between relapses [42]. The disease can also be characterized by two types of onset patterns—an apparent and acute onset that patients usually remember, or a more gradual onset with slow progression and gradual worsening of the symptoms [43].

### 3.4. Triggering Agents and Pathogenesis

The etiology of ME/CFS remains poorly understood, which results in a lack of proper diagnosis and effective treatments [44]. It is suggested that ME/CFS has a multifactorial origin due to the heterogeneity of patients and the variety of its clinical manifestations [45]. Recent discoveries have shed light on the onset mechanisms of ME/CFS, which are assumed to be via viral infections, immune dysfunctions, hypothalamic–pituitary–adrenal (HPA) system abnormalities, oxidative stress, impaired oxidative phosphorylation, and a pro-inflammatory gut microbiome [46,47,48].

Since many ME/CFS cases develop after an infection, especially a viral one, some physicians describe it as post-viral chronic fatigue [44]. Several infectious agents—most of them capable of causing lifelong latency and infecting the nervous system—have been proposed as potential triggers of ME/CFS. These include Epstein–Barr Virus, Ross River virus, human herpesvirus-6, human cytomegalovirus, parvovirus B19, human retroviruses, and enteroviruses [12]. The recent pandemic of COVID-19 infection has renewed interest in post-viral syndromes and their link to ME/CFS. New evidence suggests that SARS-CoV-2 can trigger a condition known as long-COVID, whose characteristics overlap with those of ME/CFS [49]. Consequently, it is not surprising that the number of new ME/CFS cases has risen dramatically over the last few years [50].

Because ME/CFS often starts after an infectious-like episode, immune system dysregulation has been suggested as one of its mechanisms of development [23,32]. Studies have implied that the alterations in the immune mechanisms lead to chronic low-grade systemic inflammation [41]. Generally, immune dysfunction in such patients is represented by changes in B and T-cell phenotypes, a reduction in natural killer cells’ cytotoxic activity, alterations in the cytokine profile, and immunoglobulin levels [51]. What is more, autoreactive immune cells capable of producing autoantibodies during common infections, such as those against β2-adrenergic and M3 acetylcholine receptors, can be present in some CFS patients [52].

Another mechanism discussed in ME/CFS pathogenesis involves a neuroendocrine impairment, which is manifested by abnormal levels of hormones produced by the HPA axis [53]. This has been associated with worsening of certain clinical manifestations, such as sleep and concentration difficulties, as well as fatigue debilitation [54]. Clinical studies have shown that HPA axis dysfunction in ME/CFS patients results in lower cortisol levels, reduced daily cortisol variation, and a weakened stress response, thereby worsening fatigue presentation [55].

Increased oxidative stress may also be one of the reasons for the development of ME/CFS. Several studies reported elevated levels of oxidative stress biomarkers, such as isoprostane, oxidized low-density lipoproteins (LDL), and iso-prostaglandin F2, together with lowered glutathione levels, indicating impaired antioxidant properties [56,57]. This imbalance between pro- and antioxidant systems can lead to the generation of reactive molecules, which further cause cellular injury, immune activation, and a state of chronic low-grade inflammation [58]. As a result, mitochondrial dysfunction and impairment of adenosine triphosphate (ATP) synthesis may follow, contributing to the onset of ME/CFS symptoms like fatigue and post-exertional malaise [56].

Although scientists have shown great interest in ME/CFS and many studies have been carried out, the cause and the development of the disease remain not fully understood, which makes diagnosis and treatment particularly challenging. It is now believed that many inflammatory and autoimmune diseases may be linked to changes in gut microbiota composition and the body’s impaired response to dysbiosis [59,60]. Disruption of gut microbiota homeostasis can also be considered as a possible factor in the development of ME/CFS [61].

## 4. Gut Microbiome and ME/CFS

### 4.1. Overview of Gut Microbiome

A recent hypothesis on ME/CFS pathogenesis is that it can be associated with microbial triggers that are not externally introduced but rather reside within the host, mainly those that are part of the intestinal microbiome [62]. The term gut microbiome represents an enormous community of microorganisms, including a variety of bacteria, viruses, fungi, and unicellular eukaryotes [63]. They have co-evolved with the human host to form a particularly beneficial relationship [64]. These microbes are known to have numerous relevant functions, one of which is their participation in our nutritional processes [65]. Apart from being able to extract energy and chemicals from our diet, this complex ecosystem plays several additional roles in human health by shaping immunity [66], protecting against pathogens [67,68] and producing biologically active compounds such as short-chain fatty acids (SCFAs), hormones, and vitamins [69]. It has been well documented that the gut microbiome also modulates various neurocognitive processes such as mood, cognition, and memory through a bidirectional communication between the GI tract and the central nervous system (CNS) known as the “gut–brain axis” [70].

Thanks to the recent advancements in genomic studies, the composition of the gut microbiome has been extensively studied with characterization of the major taxonomic groups of *Firmicutes*, *Bacteroides*, *Proteobacteria*, *Fusobacteria*, *Verrucomicrobia*, *Cyanobacteria*, and *Actinobacteria* residing within the intestinal tract [71]. The gut microbiome is characterized by high diversity in every human, meaning that no standard or normal microbiome can be described [50]. Its composition is influenced by various factors such as diet and lifestyle habits, genetic factors, environmental exposures, medical treatments, and even stress [72]. This indicates that the gut ecosystem is a plastic entity and can change in certain circumstances [73].

Disruption of this microbial ecosystem, which is known as gut dysbiosis, is nowadays thought to contribute to the development of many intestinal (IBS, IBD, and colorectal cancer) and extraintestinal (obesity, type 2 diabetes, and CNS-related disorders) disorders [71]. As many ME/CFS patients complain of GI symptoms, and there are frequent comorbidities with IBS [74] and IBD [75], more and more studies are focused on investigating any potential link between intestinal dysbiosis and disease severity and progression.

### 4.2. Gut Microbiota Differences in ME/CFS Patients Compared to Healthy Controls (HC)

In recent years, several studies have demonstrated shifts in gut microbiome composition in ME/CFS patients when compared to healthy individuals, with implications of dysbiosis involvement in disease pathogenesis [76,77,78,79,80,81,82,83,84,85,86,87,88]. Nevertheless, no specific microbiome pattern in ME/CFS has been detected yet [62]. This variation may reflect differences in study designs, including sample size, recruitment criteria, and methods used to characterize the intestinal microbiome composition. Furthermore, a lot of ME/CFS patients take various medications to manage their diverse symptoms, and many of these drugs (not only antibiotics) can alter the microbiome [41]. All of these findings suggest that a standardized protocol for investigating the composition of gut microbiome in ME/CFS patients is still lacking, and that is why appropriate comparisons between studies cannot be made [89].

However, when examining the articles individually, the following statistically significant changes in the relative abundance of certain taxa between the microbiomes of ME/CFS patients compared to HC can be listed (see Table 1). Most noticeably, in ME/CFS patients, the proportion of Firmicutes is reduced, accompanied by an increase in Bacteroidetes [77,78,79,84]. Authors refer to this as an overall lower *Bacteroidetes*/*Firmicutes* ratio. Usually, a lower *Bacteroidetes*/*Firmicutes* ratio is accompanied by a rise in *Enterobacteriaceae* abundance, which suggests an entirely remodeled composition of the gut microbiome [90]. Another important finding is that several bacterial genera with anti-inflammatory functions, such as *Faecalibacterium*, *Bifidobacterium*, and *Roseburia*, are decreased in the group of ME/CFS patients [21]. Furthermore, the decrease in *Faecalibacteruim prausnitzii* is considered a potential biomarker with diagnostic value in ME/CFS [81]. *Faecalibacterium* and *Bifidobacterium* are well known for their immunomodulatory functions due to production of the SCFA butyrate. Butyrate is a major source of energy for colon epithelial cells and is one of the anti-inflammatory metabolites that maintains the integrity of mucosal barriers [45,91,92]. Thus, a deficiency in this metabolite can contribute to a variety of physiological disturbances, including a weakened intestinal barrier, microbial translocation, and increased production of pro-inflammatory cytokines. This further leads to chronic inflammation, which in the case of ME/CFS, is implicated in the development of fatigue, neurocognitive, and GI symptoms [40]. A single study also noted reduced levels of *Anaerostipes*, which belongs to the family of *Lachnospiraceae* and is also a butyrate producer in the intestines [84]. An increase in the abundance of *Alistipes* was noted in ME/CFS patients from two studies [77,81]. Although *Alistipes* may have protective effects in disease, including colitis, liver fibrosis, and cardiovascular disease, it has been shown that these bacteria can also play a pathogenic role in anxiety, depression, and chronic fatigue syndrome [93]. A paper by Kitami et al. showed that the abundances of *Coprobacillus*, *Eggerthella*, and *Blautia* were the strongest indicators that could be used for microbiome differentiation between ME/CFS patients and HC [83]. The only study that harbored standard culture techniques for bacterial identification reported increased levels of D-lactic acid-producing bacteria from *Streptococcus* and *Enterococcus* spp., contributing to neurological impairment and cognitive dysfunction in these patients [76].

Despite the available findings showing inconsistency, there is enough evidence of alterations in the gut microbiome in ME/CFS patients, as noted in previous reviews [21,41,94]. In addition, many affected individuals report experiencing GI disturbances, including pain, discomfort, and an alteration in bowel habits [61]. However, the exact role of gut dysbiosis in the pathogenesis is still a matter of debate, and it cannot be said whether these alterations precede, cause, or are a consequence of immunologic or metabolic changes that accompany ME/CFS [23]. This question should be investigated in further studies, involving more participants recruited according to strict diagnostic criteria, and using standardized methods to study gut microbiome composition.

### 4.3. Mechanisms That Link Gut Dysbiosis to ME/CFS Pathogenesis

A potential link between the pathophysiology of ME/CFS and alterations in the gut microbiome can be based on the communication that exists between the GI tract and the CNS [61]. This bidirectional network, known as the microbiota–gut–brain axis, is vital for homeostasis and has been studied since the nineteenth century [70,95]. Neural, immune, and endocrine pathways are involved in the communication between the gut and the brain [96]. Direct signaling occurs mainly through the vagus nerve, while indirect signaling includes the HPA axis, immune-derived cytokines, the metabolic processing of tryptophan (TRP), and microbially derived neurotransmitters generated by some bacterial species inhabiting the gut [97,98].

Improper functioning of this axis has been implicated in the development of various disorders such as neurodegenerative conditions, including Parkinson’s disease, Alzheimer’s disease, multiple sclerosis, and autism, thus suggesting a possible role in disease pathogenesis [98,99,100]. Focusing on ME/CFS, dysfunction of the gut–brain axis may be responsible for symptoms related to impaired neurocognition, sleep, and mood changes that are frequently reported by patients [101].

**Figure 2:**
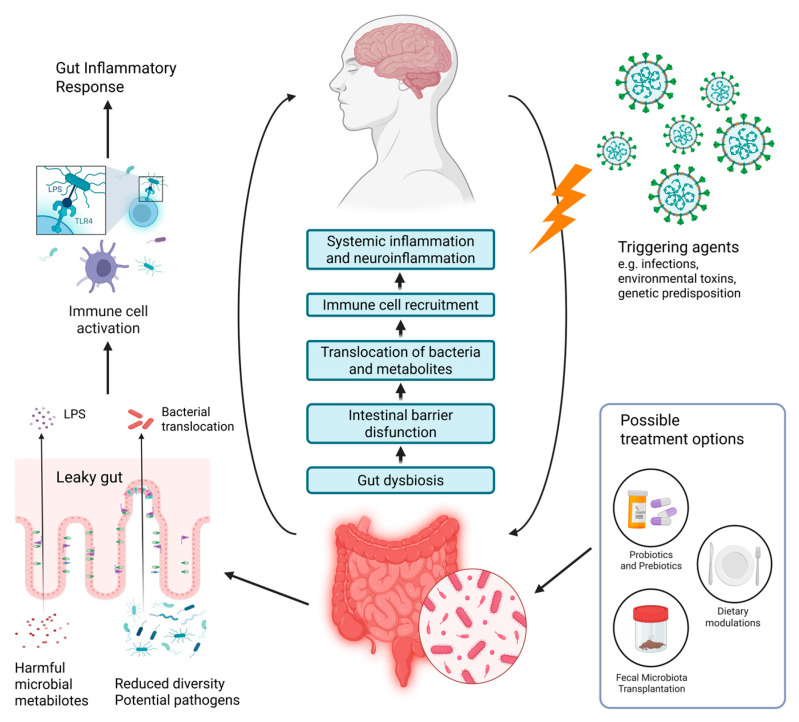
Overview of the gut–brain axis in the development of ME/CFS and potential treatment options based on intestinal microbiome modulation (modified after [106]). Created in BioRender. Nikolova, R. (2025) https://BioRender.com/t3aaxo1, accessed on 1 November 2025.

Despite the lack of detailed knowledge on the mechanisms involving the gut–brain axis, several theories attempt to give an explanation for the interplay between gut dysbiosis and ME/CFS symptomatology (Figure 2). Recent studies suggest that intestinal dysbiosis may trigger a series of biological responses, which include increased intestinal permeability, translocation of microbes or their metabolic products (i.e., lipopolysaccharides (LPS)), activation of immune responses, and cytokine release leading to gut inflammation and further worsening of the intestinal barrier permeability [41,50,90]. In addition, some of the microbial metabolites act as neurotransmitters that alter the signaling along the gut–brain axis [61,62].

When the intestinal barrier functions normally, harmful substances like bacteria and toxins are prevented from crossing the intestinal epithelium and reaching the body [93]. The presence of a compromised intestinal barrier known as ‘leaky gut’ is supported by several studies that have found higher levels of LPS and antibodies against LPS in the peripheral blood of ME/CFS patients than in controls [75,78,102,103]. The increased presence of Gram-negative bacteria associated with dysbiosis may be the main reason for the elevated amount of LPS in the circulation [79]. This is known as metabolic endotoxemia, and according to Giloteaux et al., increased endotoxin levels disrupt the epithelial barrier, enter the circulation and activate the inflammatory TLR-4 pathway [79,104,105]. Consequently, pro-inflammatory cytokines (IL-1 and TNF-α) as well as reactive oxygen species are being produced by immune cells, thus enhancing systemic inflammation and symptom severity [103]. Since the translocation of bacteria or their products can induce systemic inflammation, alter the blood–brain barrier, and trigger neuroinflammation, some researchers believe that this process may be the basis for neurological disturbances in ME/CFS patients. This suggests that therapies aimed at reducing intestinal permeability may improve both GI and cognitive manifestations, but more research is required [90].

Another potential finding that can enlighten the consequences of dysbiosis in ME/CFS patients is the reduced abundance of butyrate-producing species *Faecalibacterium prausnitzii* and *Eubacterium rectale* [86]. As previously mentioned, SCFA such as butyrate are important for mucosal integrity and immune modulation [45]. A study has shown that higher fecal butyrate levels are linked to greater physical fitness, as well as increased gut microbial diversity and lower LPS synthesis [107]. Thus, the decrease in SCFA can disrupt the normal barrier function of the intestinal epithelium and promote intestinal inflammation [86].

Gut microbiome changes due to the use of antibiotics have been recently hypothesized in ME/CFS development, as antibiotics are known to alter the bacterial composition in the gut for up to four years, and in some cases, the changes may never fully recover [108]. There are no specific studies on the direct influence of antibiotics as potential triggers of ME/CFS, but it is known that these patients suffer from frequent infections, which is probably associated with higher use of antibiotics in these patients [41]. Possible explanations for the effects of antibiotic intake in ME/CFS include gut microbiome alterations, leading to reduced production of anti-inflammatory metabolites or promoting an environment that favors the growth of D-lactate-producing bacteria [94]. However, further studies are needed to evaluate the impact of antibiotic intake on microbiome composition and ME/CFS development.

Neurotransmitters like serotonin and dopamine seem to play an enormous role in gut–brain axis functioning [109,110]. This led researchers to explore whether changes in the balance of these neuromodulators can cause chronic fatigue [111]. Since most serotonin comes from the TRP metabolism in the gut, and serotonin levels in blood do not reflect those in the brain, studies now focus on TRP metabolites, especially those that are produced through the kynurenine pathway involving indoleamine 2,3-dioxygenase (IDO) [112]. Pro-inflammatory cytokines can activate IDO, resulting in elevated production of kynurenine, which is important for immune tolerance [113]. Nevertheless, the results concerning these metabolites in ME/CFS patients are conflicting. Some researchers hypothesize that there is insufficient kynurenine production from TRP in ME/CFS patients, resulting in disturbances of the CNS, GI, and immune system [114]. High TRP and low kynurenine levels are thought to interfere with serotonin and melatonin pathways and contribute to ME/CFS symptom development. On the other hand, high amounts of kynurenine and some of its metabolites are observed in some diseases whose symptoms, such as higher sensitivity to pain, light, and sound, overlap with those of ME/CFS [41]. As TRP is also metabolized by gut bacteria, dysbiosis can influence the production of TRP- and phenylalanine-derived metabolites, potentially affecting the gut, brain, and the microbiota–gut–brain axis [115]. If the exact mechanisms of the kynurenine pathway are thoroughly studied, this may lay the foundations of new therapeutic targets in ME/CFS.

### 4.4. Moving Forward—New Directions for ME/CFS and Microbiome Studies

Most reviews and future directions in ME/CFS microbiome research highlight important but already acknowledged needs and suggestions, such as larger cohorts, improved standardization of sampling and sequencing methodologies, and longitudinal studies to track microbiome dynamics [22,41,87,94,116]. These remain essential steps toward more reliable and comparable results. However, a less recognized area of research is the integration of host genetic information into microbiome studies. The inclusion of single nucleotide polymorphism (SNPs) of patients’ own genomes, or at least the use of existing data from genome-wide association studies (GWAS), would allow researchers to use approaches such as Mendelian randomization (MR) to draw conclusions about causality. This approach could highlight whether there are specific changes in the microbiome that play a role in the development of ME/CFS or are rather a consequence of the disease itself. Although adding genetic data on the host would undoubtedly make research more expensive and analysis more difficult, it represents a realistic and valuable direction for moving from purely associative discoveries to a more definitive insight into the biological causation of ME/CFS.

## 5. Microbiome Modulation Therapies as Treatment Strategy of ME/CFS

Microbiome disturbances in ME/CFS patients are characterized by reduced microbial diversity and an imbalance between bacteria with pro- and anti-inflammatory functions, which further cause increased gut permeability and chronic inflammation in multiple organs [79,94]. These findings propose that therapeutic strategies such as probiotics or prebiotics supplementation, dietary interventions, and fecal microbiota transplantation (FMT) can be used to rebalance the gut microbiome and relieve the symptoms of ME/CFS [42].

### 5.1. Probiotics

The administration of potentially beneficial bacteria known as probiotics may positively influence digestion processes and immunity [61]. By modulating the immune signaling pathways and improving the functions of gut microbiota within the gut–brain axis, probiotics have been shown to reduce inflammation and oxidative stress in ME/CFS patients, thus influencing anxiety and cognitive issues [117,118]. Similar benefits were observed in a small group of ME/CFS patients who were given probiotics containing *Bifidobacterium* and *Lactobacillus*, but no meaningful changes in fatigue levels and physical activity were noted [119]. Another study showed promising results of probiotics’ ability to reduce systemic markers of inflammation, including C-reactive protein (CRP), TNF-α, and IL-6 [120]. Although probiotics have shown several beneficial effects, making them a potential treatment option for ME/CFS, the limited number of studies and some inconsistencies in findings highlight the need for further research in that area [41].

### 5.2. Prebiotics

Prebiotics such as fructo-oligosaccharides and galacto-oligosaccharides serve as nutrients for gut bacteria. They can enhance the growth of some beneficial bacterial populations like *Bifidobacterium* spp., consequently correcting microbiota imbalance [121]. What is more, out of these compounds, bacteria are able to produce SCFAs, which have local and extra-intestinal functions in the host [122]. SCFAs are known to assist in the control of intestinal inflammation in terms of gut barrier maintenance [123]. Prebiotics seem a reasonable treatment opportunity in ME/CFS, as studies with rodents fed with prebiotics show reduced gut inflammation with a presence of lower levels of LPS and pro-inflammatory cytokines in the serum [124,125].

### 5.3. Fecal Microbiota Transplantation

A procedure involving the transfer of fecal matter from a healthy donor into a recipient’s GI tract to influence dysbiosis is known as FMT [126]. It may appear as an emerging therapeutic option for a wide range of diseases, but up to now, it has been officially approved for the treatment of recurrent *Clostridioides difficile* infections [127]. FMT is also a promising treatment strategy for ME/CFS, as there are indications that the microbiome may have an enormous influence on the disease, particularly its neurological symptoms [94]. For example, a study by Kenyon et al. compared the effect of FMT to oral treatment with pro- and prebiotics in 42 patients suffering from ME/CFS with IBS comorbidity. Among the 21 patients who received FMT, 17 reported improvements, and 7 patients claimed to have a complete restoration of quality of life and daily functioning [128]. Contrary to that, another study proved that FMT was a safe treatment option, but it did not manage to relieve symptoms or improve the quality of life of those affected by ME/CFS [129]. Still, numerous limitations in FMT, including the lack of standardized protocols about administration routes, patient selection criteria, and therapy duration, have to be addressed. Although further research is needed, FMT for dysbiosis-related diseases holds potential as a novel treatment strategy for ME/CFS [90].

### 5.4. Dietary Interventions

Last but not least, dietary interventions can be one of the major modulators of the gut microbiome [74]. Western-style diets based on processed foods and saturated fats seem to promote dysbiosis and accelerated aging [130]. On the other hand, the Mediterranean diet is known for its crucial role in enhancing beneficial gut microbes and their metabolic functions [131]. Recent studies have shown that special dietary regimens such as a very-low-calorie diet and a gluten-free diet contributed to microbiota modulation, which further resulted in a reduction of immune-mediated intestinal inflammation, intestinal permeability and lower levels of circulating inflammatory markers (CRP and lipopolysaccharide-binding protein) in obesity and IBS [132,133]. Another study done on mouse models suggested that the use of Astragalus polysaccharide derived from the dry root of *Astragalus membranaceus* can be useful in alleviating CFS symptoms. The use of Astragalus polysaccharide increased the SCFA levels (especially butyrate) by regulating the gut microbiota, thus further influencing the oxidative stress and inflammation in the brain [134]. Polyphenols and omega-3 fatty acids have been proven to be beneficial in gut–brain axis modulation for the treatment of neuroinflammatory disease [135]. On the other hand, a systematic review showed insufficient evidence that ME/CFS patients may benefit from elimination or modified diets for symptom relief [136]. More clinical trials will help to unravel the potential of dietary interventions as an option for ME/CFS treatment.

## 6. Conclusions

Various studies have been performed on ME/CFS patients in the hope of finding a disease-specific microbiome signature. However, although clear evidence for shifts in bacterial populations within the gut is present, studies yield contradicting results that are hard to compare. It is still a matter of debate whether microbiome alterations precede or are a consequence of immunometabolic alterations in the disease’s course. Future studies on larger cohorts using standardized diagnostic criteria, protocols, and techniques to reduce the confounding variables of factors that influence gut microbiota composition should be adopted. Clarifying the key pathophysiological mechanisms driven by an altered microbiome in ME/CFS will support the development of therapeutic strategies aimed at modulating the gut microbiome and alleviating symptoms.

## Figures and Tables

**Table 1:** Microbiome composition differences between individuals with ME/CFS and healthy controls (HC).

Study (Year)	Subjects	Diagnostic Criteria	Microbiome Analysis Method	Main Findings
Sheedy et al., 2009 [76]	285 (108 ME/CFS, 177 HC)	Holmes, Fukuda and Canadian Criteria	Culture methods	↑ *Enterococcus* and *Streptococcus* spp.
Frémont et al., 2013 [77]	79 (43 ME/CFS, 36 HC)	Fukuda Criteria	High-throughput 16S rRNA gene sequencing	↑ *Lactonifactor* and *Alistipes*; ↓ *Firmicutes* (several populations)
Shukla et al., 2015 [78]	20 (10 ME/CFS, 10 HC)	Fukuda Criteria	16S rRNA gene sequencing	↑ *Bacteroidetes* (non-significant); ↓ *Actinobacteria*; *Firmicutes* (non-significant)
Giloteaux et al., 2016 [79]	87 (48 ME/CFS, 39 HC)	Fukuda Criteria	High-throughput 16S rRNA gene sequencing	↑ *Proteobacteria* species (*Enterobacteriaceae*); ↓ *Firmicutes*
Giloteaux et al., 2016 [80]	2 (1 ME/CFS and 1 HC); A pair of 34-year-old monozygotic twins	Fukuda Criteria	High-throughput 16S rRNA gene sequencing	↓ Microbial diversity; ↓ *Faecalibacterium* and *Bifidobacterium*;
Nagy-Szakal et al., 2017 [81]	100 (50 ME/CFS, 50 HC)	Fukuda, Canadian Criteria	Shotgun metagenomic sequencing	Patients with ME/CFS: ↑ *Bacteroides*; ↓ *Bacteroides vulgatus*; Patients with ME/CFS and IBS: ↑ *Alistipes*; ↓ *Faecalibacterium*
Mandarano et al., 2018 [82]	88 (49 ME/CFS, 39 HC)	Fukuda Criteria	18S rRNA sequencing	↑ *Basidiomycota*/*Ascomycota* ratio (non-significant); ↓ eukaryotic diversity (non-significant)
Kitami et al., 2020 [83]	100 (48 ME/CFS, 52 HC)	Fukuda and International Consensus Criteria	High-throughput 16S rRNA gene sequencing	↑ *Coprobacillus*, *Eggerthella* and *Blautia*; ↓ *Faecalibacterium*
Lupo et al., 2021 [84]	105 (35 ME/CFS, 70 HC)	Fukuda Criteria	16S rRNA gene sequencing	↑ *Bacteroides* and *Phascolarctobacterium*; ↓ *Lachnospiraceae* (*Anaerostipes*)
Xiong et al., 2023 [85]	228 (149 ME/CFS, 79 HC)	Fukuda, Canadian and Institute of Medicine Criteria	Shotgun metagenomic sequencing	↓ *Roseburia*, *F. prausnitzii* ↓ Microbial diversity; altered *Bacteroidetes*/*Firmicutes* ratio
Guo et al., 2023 [86]	197 (106 ME/CFS, 91 HC)	Fukuda and Canadian Criteria	Shotgun metagenomic sequencing	↑ *Clostridium bolteae*, *Ruminococcus gnavus;* ↓ *Faecalibacterium prausnitzii*, *Eubacterium rectale*
He et al., 2023 [87]	462,933 (2076 ME/CFS, 460,857 HC)	-	GWAS, MR	↑ Anaerobic bacteria (*Paraprevotella*, *Ruminococcaceae UCG_014*)
Prylinska-Jaskowiak et al., 2025 [88]	41 (25 ME/CFS, 16 HC)	Fukuda criteria	16S rRNA gene sequencing	↑ *Bacteroidetes* (*Bacteroidaceae*); ↓ *Firmicutes* (*Lachnospiraceae* and *Veillonellaceae*)

↑ increased amount in ME/CFS; ↓ decreased amount in ME/CFS; rRNA—ribosomal RNA; IBS—irritable bowel syndrome; GWAS—Genome-wide association study; and MR—Mendelian randomization.

## Data Availability

No new data were created or analyzed in this study. Data sharing is not applicable to this article.
